# Knowledge, attitudes, and practices related to COVID-19 among patients at *Hospital Universitario de Caracas* triage tent: A cross-sectional study

**DOI:** 10.7705/biomedica.5808

**Published:** 2021-10-15

**Authors:** Fabián R. Chacón, Juan M. Doval, Verónica I. Rodríguez, Adriana Quintero, Daniela L. Mendoza, Mario D. Mejía, Óscar D. Omaña, Mariana B. Contreras, Sebastián Gasparini, Carmen J. González, Natasha A. Camejo-Ávila, Sinibaldo R. Romero, David M. Flora-Noda, Andrea L. Maricuto, Viledy L. Velásquez, Rafael N. Guevara, Martín Carballo, Jocays Caldera, María C. Redondo, María E. Landaeta, Fhabián S. Carrión-Nessi, David Alberto Forero-Peña

**Affiliations:** 1 Escuela de Medicina, Universidad Central de Venezuela, Caracas, Distrito Capital, Venezuela Universidad Central de Venezuela Universidad Central de Venezuela Caracas Distrito Capital Venezuela; 2 Instituto de Investigación Biomédica y Vacunas Terapéuticas, Ciudad Bolívar, Venezuela Instituto de Investigación Biomédica y Vacunas Terapéuticas Ciudad Bolívar Venezuela; 3 Medical Scientist Training Program (MD/PhD), University of Minnesota Medical School, Minneapolis, Minnesota, United States University of Minnesota University of Minnesota Medical School Minneapolis Minnesota USA; 4 Departamento de Enfermedades Infecciosas del Adulto, Hospital Universitario de Caracas, Caracas, Distrito Capital, Venezuela Hospital Universitario de Caracas Caracas Distrito Capital Venezuela

**Keywords:** coronavirus infections, health knowledge, attitudes, practice, health promotion, Venezuela, infecciones por coronavirus, conocimientos, actitudes y práctica en salud, promoción de la salud, Venezuela

## Abstract

**Introduction:**

The studies on knowledge, attitudes, and practices (KAP) regarding COVID-19 help to identify erroneous concepts and inadequate practices related to the disease. This baseline information is essential to design effective strategies and improve adherence to prevention measures.

**Objective:**

To identify the COVID-19-related KAP in Venezuelan patients screened at the *Hospital Universitario de Caracas* triage tent.

**Materials and methods:**

We conducted a cross-sectional study among 215 patients between April 25^th^ and May 25^th^, 2020, with in-person interviews using a KAP survey.

**Results:**

Most surveyed patients (53.5%) were asymptomatic. Most of them, both from the symptomatic and the asymptomatic groups, had adequate knowledge about the symptoms and transmission of the disease and the majority said they were practicing quarantine, frequent handwashing, and the use of face masks in public areas. However, the daily replacement of cloth face masks was more frequent in the asymptomatic group whereas replacement every three days was more frequent in the symptomatic group. Finally, more than half of the participants admitted having been in crowded places, a common practice among the symptomatic compared to the asymptomatic patients.

**Conclusions:**

This is the first KAP study in Venezuela about COVID-19. Knowledge and practices among Venezuelans could be improved by strengthening education and training programs. This information from the early phase of the pandemic in Venezuela may contribute to the design of COVID-19 promotion and prevention strategies.

## Resumen

**Introducción.:**

Los estudios de conocimientos, actitudes y prácticas (CAP) relacionados con la Covid-19 permiten establecer los conceptos erróneos y las prácticas inadecuadas en torno a esta enfermedad, información fundamental para diseñar estrategias eficaces y mejorar el cumplimiento de las medidas de prevención.

**Objetivo.:**

Establecer los conocimientos, actitudes y prácticas relacionadas con la Covid-19 en pacientes venezolanos cribados en la carpa de triaje del Hospital Universitario de Caracas.

**Materiales y métodos.:**

Se hizo un estudio transversal en 215 pacientes entre el 25 de abril y el 25 de mayo de 2020, mediante entrevistas personales y utilizando una encuesta sobre conocimientos, actitudes y prácticas.

**Resultados.:**

La mayoría (53,5 %) de los pacientes encuestados se encontraba asintomática; los encuestados tenían un conocimiento adecuado sobre los síntomas y la transmisión de la enfermedad, sin distinción entre sintomáticos y asintomáticos. La mayoría de ellos indicó estar respetando las prácticas de cuarentena, lavado de manos frecuente y uso de tapabocas en público; sin embargo, el reemplazo diario de los tapabocas de tela fue más frecuente en el grupo de los asintomáticos, en tanto que el reemplazo cada tres días fue más común entre los sintomáticos. Por último, más de la mitad de los participantes admitió haber estado en lugares concurridos, siendo esta práctica más frecuente entre los sintomáticos que entre los asintomáticos.

**Conclusiones.:**

Este es el primer estudio de conocimientos, actitudes y prácticas sobre la Covid-19 en Venezuela. El conocimiento y las prácticas de los venezolanos podrían mejorar mediante el fortalecimiento de los programas de educación y capacitación. Esta información, obtenida durante la fase inicial de la pandemia en Venezuela, podría contribuir al diseño de estrategias de promoción y prevención de la Covid-19.

The severe acute respiratory syndrome coronavirus 2 (SARS-CoV-2) causes the now called coronavirus disease 2019 (COVID-19) first reported in December 2019, in Wuhan, China ^1^. The World Health Organization (WHO) declared COVID-19 to be an international pandemic on March 11^th^, 2020 ^2^. Currently, the disease has spread to more than 190 countries with 126,372,442 cases and 2,769,696 deaths around the world ^3^. Despite efforts to mitigate it, the number of cases continues to rise in Latin America and the Caribbean ^4^. Throughout the American continent, every country has reported cases of COVID-19 with Brazil, the United States, Perú, Argentina, Mexico, and Colombia as the most affected ones. In Venezuela, by the time this study was made (May 25^th^, 2020), the number of cases was 1,211, and deaths summed up to 10. Until March 21^st^, 2021 ^5^, more than a year after the confinement, the number of cases was 149,145 and deaths amounted to 1,475 according to WHO ^3^ while the country’s Ministry of Health had reported 155,663 cases and 1,555 deaths until March 29^th^, 2021.

By March 24^th^, 2021, three SARS-CoV-2 variants of concern, classified by WHO as B.1.1.7, B.1.351, and B.1.1.28, had been identified possibly increasing transmissibility, virulence, or a detrimental change in COVID-19 epidemiology or clinical presentation. In the region, variants of interest have also been reported in Argentina, Chile, México, Saint Maarten, the United States, Uruguay, and Venezuela, among them, the Brazilian variant P.2 whose proportion increased in this country from 0.7% to 45% between September 2020 and February 2021 ^6^.

On March 11^th^, 2020, a 41-year-old female was admitted to *Hospital Universitario de Caracas* (HUC) with a three-day story of fever, headache, and runny nose. She had traveled to Spain and Italy. The patient’s nasopharyngeal swab tested positive for SARS-CoV-2 by reverse transcriptase-polymerase chain reaction (RT-PCR), thus confirming the first COVID-19 case in Venezuela (unpublished data). Immediately, the HUC installed a triage tent attached to the Infectious Diseases Department to evaluate suspected cases of COVID-19. Healthcare authorities and the Venezuelan Ministry of Health declared the HUC as the main satellite hospital for the screening and management of SARS-CoV-2 infection.

On March 13^th^, 2020, the state of national emergency was decreed in Venezuela, and measures of social distancing and the mandatory use of masks in public spaces were established and three days after, a national quarantine was declared ^7^. Despite these efforts, COVID-19 rapidly spread throughout the country. To maintain the number of cases at a minimum, it is essential that the public adheres to the sanitary control measures established by national and international authorities. The knowledge, attitudes, and practices (KAP) related to COVID-19 identify the willingness of the population to accept the behavioral changes implicit in the measures ^8^. KAP surveys measure individuals’ knowledge and describe their attitudes and practices. Nevertheless, knowledge does not necessarily translate into the adoption of good practices. Which affects the population’s adherence to the control measures required to contain the spread of COVID-19.

KAP studies related to COVID-19 provide baseline information and direct insight about the disease, and contribute to the development of interventions targeting misconceptions and inadequate practices, as well as identifying strategies to improve the attitude of the population towards the pandemic control. In Latin America, there are few KAP studies about COVID-19 ^10^^,^^11^ and to the best of our knowledge, there are none in Venezuela. In this context, we conducted a cross-sectional study to identify KAP related to COVID-19 among Venezuelan patients screened at the HUC triage tent.

## Materials and methods

### Study design and population

A cross-sectional survey was conducted among patients screened at the triage tent attached to the HUC Infectious Diseases Department between April 25^th^ and May 25^th^, 2020. Patients were included regardless of their consultation motive, or the presence of symptoms, or epidemiological link. The questionnaire required five minutes to be completed. The sample size was calculated using the population formula ^12^ and taking into account the number of individuals evaluated in the triage tent the previous month. A minimal sample size of 208 respondents was obtained with a 95% confidence interval and a 5% margin of error. Consecutive patients were invited to participate; we explained the objectives of the study and upon their acceptance, we asked them to answer a survey applied in a face-to-face interview by trained researchers at the HUC.

### Measures

The survey was designed based on WHO recommendations and previous KAP studies in other countries ^13^^-^^15^. A group of clinicians with expertise in infectious diseases and epidemiology assessed the survey instrument and provided insight on its relevance and accuracy, as well as on the instrument simplicity keeping in mind the study population. The questionnaire was pretested on 30 participants in a pilot study. The data generated from this initial study was excluded from the final analyses.

The survey had a total of 34 questions: eight on socio-demographic characteristics including identification, gender, age, place of origin, address, occupation, education, and ethnic identity; two related to symptoms and epidemiological link; nine about previous knowledge on COVID-19 symptoms, transmission, and prevention; four about attitudes towards COVID-19 control and prevention, and eight questions about practices such as handwashing, social distancing, and wearing face masks, among others. In addition, three questions were included for occupation, educational level, and ethnicity.

### Statistical analysis

The data analysis included descriptions of the characteristics of the sample studied using central tendency and variability measures (average, standard deviation) for the age variable, which had a normal distribution (Kolmogorov-Smirnov). The other variables were analyzed using Pearson’s chi-square test, chi-square test with Yates’ correction, and Fisher’s exact test as appropriate. A *p*-value < 0.05 was considered significant. The data were processed using SPSS v.25.0. Binomial and multinomial logistic regression analyses were performed to determine the relationships between sociodemographic characteristics, the presence or absence of symptoms, and KAP; the variables included were age, sex, occupation, education, the reason for consultation, and the presence or absence of symptoms.

## Ethical considerations

The study was conducted in accordance with the Declaration of Helsinki (WMA, 2013). The protocol was reviewed and approved by the *Centro Nacional de Bioética* (CENABI), Caracas, Venezuela. The participation in the survey was anonymous, consensual, and voluntary, with informed consent given by all respondents.

## Results

We surveyed 215 patients screened at the HUC triage tent. Most of them (53.5%) were asymptomatic while 100 (46.5%) were symptomatic. Only 7.9% of the patients had an epidemiological link (travel history to a high transmission area or exposure to a suspected or confirmed COVID-19 case). All patients evaluated in the study had a negative RT-PCR. Table 1 shows additional detailed socio-demographic characteristics of the sample.

**Table 1:** Socio-demographic characteristics of the patients

Characteristics	Total n = 215 (100%)	Symptomatic n = 100 (46.5%)	Asymptomatic n = 115 (53.5%)	*p-value*
Age, years (mean)	40 (15)	41 (15)	39 (16)	0.258*
Sex			
Male	111 (51.6)	56 (56)	55 (47.8)	0.232^†^
Female	104 (48.4)	44 (44)	60 (52.2)	
Occupation (sector)				
Merchant	35 (16.3)	17 (17)	18 (15.7)	
Student	28 (13)	10 (10)	18 (15.7)	
Health care worker	23 (10.7)	12 (12)	11 (9.6)	
Administrative worker	19 (8.8)	10 (10)	9 (7.8)	
Stay at home spouse	18 (8.4)	10 (10)	8 (7)	0.062^†^
Food sector	10 (4.7)	5 (5)	5 (4.3)	
Workman/Maintenance	15 (7)	8 (8)	7 (6.1)	
Telephone operator	6 (2.8)	0 (0)	6 (5.2)	
Other	26 (12.1)	9 (9)	17 (14.8)	
Security guard/Military/Policeman	19 (8.8)	14 (14)	5 (4.3)	
Transportation	8 (3.7)	1(1)	7 (6.1)	
Educational level			
Primary education	49 (22.8)	28 (28)	21 (18.3)	
Secondary education	89 (41.4)	40 (40)	49 (42.6)	0.109^‡^
University/Technical	75 (34.9)	30 (30)	45 (39.1)	
None	2 (0.9)	2 (2)	-	
Main consultation motive				
I need the screening test to travel.	66 (30.7)	3 (3)	63 (54.8)	
I think I have the symptoms of COVID-19.	52 (24.2)	51 (51)	1 (0.9)	
I had contact with a confirmed or suspected COVID-19 case.	52 (24.2)	51 (51)	1 (0.9)	0.000^‡||^
My family/doctor/employer told me to come to take the test to rule out	74 (34.4)	33 (33)	41 (35.7)	
I have a comorbidity.	2 (0.9)	1 (1)	1 (0.9)	
I am afraid of COVID-19.	8 (3.7)	6 (6)	2 (1.7)	
I live with vulnerable people such as elders or people with comorbidities.	3 (1.4)	3 (3)	0 (0)	

^*^ t-Student

^†^ Chi^2^ Pearson

^‡^ Exact Fisher test

^|^ ^|^ Post-hoc analysis only significant for asymptomatic and “I think I have the symptoms of COVID-19” (standardized residues: 5.5) and “I need the screening test to travel” (standardized residues: 4.7)

^¶^ Post-hoc analysis only significant for symptomatic and ‘The doctor told me to come to rule out coronavirus” (standardized residues: 2.5)

^* *^ Post-hoc analysis only significant for asymptomatic and “Trip to other state or country” (standardized residues: 4.7)

**Figure 1:**
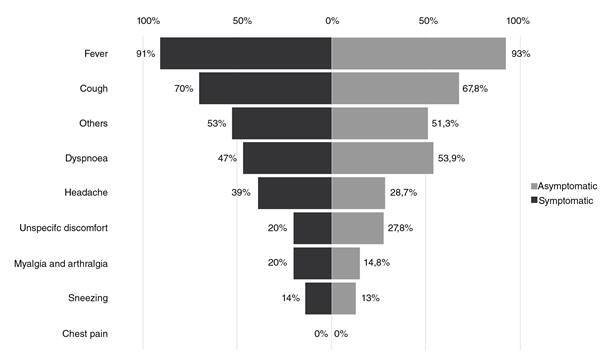
Knowledge about COVID-19 symptoms

Regarding patients’ knowledge, fever, cough, and dyspnea were identified as the first symptoms detected in COVID-19 patients by both symptomatic and asymptomatic groups (figure 1). The most frequently recognized transmission mechanism was by droplets and secretions (75.3%) followed by physical contact (56.7%). The most frequent preventive measures known by patients were wearing face masks in public areas (79.5%), frequent handwashing practices (66.5%), and self-quarantine (50.7%) with no differences between the two groups (table 2). More than half of the interviewees (53%) believed that Venezuela lacks enough resources to control the COVID-19 infection. Almost all the interviewees (96.7%) agreed that quarantine compliance could decrease the number of cases and most said (83.3%) that the national quarantine should continue in Venezuela. Finally, most patients (83.3%) felt that doctors should not work without personal protection equipment.

*p* > 0.05 (Chi^2^ Pearson)

Others include sore throat, runny nose, nasal congestion, diarrhea.

**Table 2:** Knowledge about COVID-19 in symptomatic and asymptomatic patients screened at the triage tent

Knowledge	Total n = 215 (100%)	Symptomatic n = 100 (46.5%)	Asymptomatic n = 115 (53.5%)	*p-value*
How does COVID-19 spread?				
By contacting with droplets and secretions from an infected person	162 (75.3)	72 (72.0)	90 (78.3)	0.288*
By maintaining physical contact with an infected person	122 (56.7)	53 (53.0)	69 (60.0)	0.301*
By entering a contaminated space from an infected person	98 (45.6)	47 (47.0)	51 (44.3)	0.697*
Other	36 (16.7)	22 (22.0)	14 (12.2)	0.054*
By maintaining sexual intercourse with an infected person	1 (0.5)	-	1 (0.9)	0.350^†^
Didn’t know/Didn’t answer	1 (0.5)	-	1 (0.9)	0.350^†^
How can COVID-19 be prevented?				
Wearing a face mask in public areas	171 (79.5)	82 (82.0)	89 (77.4)	0.403*
Hand washing frequently	143 (66.5)	69 (69,0)	74 (64.3)	0.562*
Respecting quarantine	109 (50.7)	47 (47.0)	62 (53.9)	0.340*
Wearing gloves in public areas	78 (36.3)	31 (31.0)	47 (40.9)	0.156*
Disinfecting and keeping the house clean	60 (27.9)	27 (27.0)	33 (28.7)	0.879*
Practicing social distancing	52 (24.2)	24 (24.0)	28 (24.3)	0.953*
Other	35 (16.3)	14 (14.0)	21 (18.3)	0.461*
What is the first thing a person should do when COVID-19 symptoms begin to show?				
Go to a doctor to have the screening test done	181 (84.2)	85 (85.0)	96 (83.5)	0.016^†^
Stay home to prevent contagion to other people	23 (10.7)	7 (7.0)	16 (13.9)	
Other (Call a doctor without leaving the house)	8 (3.7)	7 (7.0)	1 (0.9)	
Didn’t know/Didn’t answer	2 (0.9)	0 (0.0)	2 (1.7)	
Take a home remedy, plant infusions, or self-medicate	1 (0.5)	1 (1.0)	-	
Can asymptomatic people infected with COVID-19 transmit the disease?				
Yes	177 (82.3)	79 (79.0)	98 (85.2)	0.044*
No	24 (11.2)	10 (10.0)	14 (12.2)	
Didn’t know/Didn’t answer	14 (6.5)	11 (11.0)	3 (2.6)	
Can COVID-19 be transmitted by pets?				
Yes	47 (21.9)	22 (22.0)	25 (21.7)	0.832*
No	143 (66.5)	65 (65.0)	78 (67.8)	
Didn’t know/Didn’t answer	25 (11.6)	13 (13.0)	12 (10.4)	
Does COVID-19 only complicate in elders or people with comorbidities?				
Yes	90 (41.9)	45 (45.0)	45 (39.1)	0.703^†^
No	123 (57.2)	54 (54.0)	69 (60.0)	
Didn’t know/Didn’t answer	2 (0.9)	1 (1.0)	1 (0.9)	
Does a cure for COVID-19 already exist?				
Yes	21 (9.8)	14 (14.0)	7 (6.1)	0.011*
No	177 (82.3)	74 (74.0)	103 (89.6)	
Didn’t know/Didn’t answer	17 (7.9)	12 (12.0)	5 (4.3)	
How or who did you learn from what you know about COVID-19?				
Traditional media sources (television, radio, posters and flyers)	159 (74.0)	77 (77.0)	82 (71.3)	^‡^
Television	155 (97.48)	75 (97.40)	80 (97.56)	
Radio	41 (25.79)	21 (27.27)	20 (24.39)	0.642*
Posters and flyers	5 (3.14)	4 (5.19)	1 (1.22)	0.319
Social media (Facebook®, Instagram®, Twitter®, WhatsApp®)	102 (47.7)	44 (44.4)	58 (50.4)	
Facebook®	58 (56.86)	31 (70.45)	27 (46.55)	0.016*
Instagram®	51 (50)	20 (90.90)	31 (53.45)	0.424*
Twitter®	33 (32.35)	13 (29.54)	20 (34.48)	0.598*
WhatsApp®	48 (47.06)	25 (56.81)	23 (39.66)	0.085*
Healthcare workers	35 (16.3)	16 (16.0)	19 (16.5)	0.918*
Friends or neighbors	18 (8.4)	9 (9.0)	9 (7.8)	0.757*
Family	10 (4.7)	8 (8.0)	2 (1.7)	0.064^‡^

* Chi^2^ Pearson

^†^ Exact Fisher test

^‡^ Chi^2^ Yates correction

**Figure 2:**
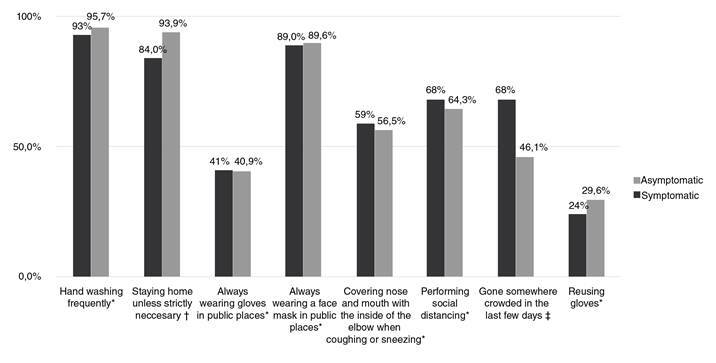
Preventive practices that the surveyed patients refer to perform

As for practices, most interviewees said that they observed preventive measures including frequent handwashing, wearing a face mask in public areas, and respecting quarantine guidelines; no differences were observed between asymptomatic and symptomatic groups (figure 2). Other less commonly preferred preventive practices are shown in table 3. More than half of the interviewees admitted to having been in a crowded place, a practice which was more frequent in the symptomatic than the asymptomatic group (68% vs. 46.1%; *p* = 0.001). In most cases (29.8%), the presence in crowded places was attributed to grocery shopping. Cloth (62.3%) and surgical type of face masks (34.9%) were the most used by the interviewees; the least frequently used (2.8%) was the N95 model. Daily mask replacement was less frequent in symptomatic patients compared to asymptomatic ones (34% vs. 56.5%; *p* = 0.002). Mask replacement every three days was more frequent among symptomatic than asymptomatic patients (46% vs. 26.1%; *p* = 0.002). Most of the interviewees (68.8%) washed their hands with soap more than five times a day (table 4).

* *p* > 0.05 (Chi^2^ Pearson) ^†^ *p*: 0.019 (Chi^2^ Pearson) ^‡^ *p*: 0.001 (Chi^2^ Pearson)

**Table 3:** Attitudes regarding COVID-19 in symptomatic and asymptomatic patients screened at the triage tent

Attitudes	n = 215 (100%)	n = 100 (46.5%)	n = 115 (53.5%)	*p-value*
Do you think Venezuela is well prepared to control the coronavirus?				
Yes	92 (42.8)	47 (47.0)	45 (39.1)	
No	114 (53.0)	50 (50.0)	64 (55.7)	
I don’t know	9 (4.2)	3 (3.0)	6 (5.2)	0.446*
Do you believe that if people respect quarantine the number of cases can lower?				
Yes	208 (96.7)	95 (95.0)	113 (98.3)	
No	7 (3.3)	5 (5.0)	2 (1.7)	
I don’t know	-	-	-	0.338^†^
Do you think that quarantine should continue?				
Yes	179 (83.3)	90 (90.0)	89 (77.4)	0.019*
No	33 (15.3)	10 (10.0)	23 (20.0)	
I don´t know	3 (1.4)	-	3 (2.6)	
In a crisis situation, do you think that doctors should take care of patients without personal protection equipment?				
Yes	34 (15.8)	19 (19.0)	15 (13.0)	0.141*
No	179 (83.3)	79 (79.0)	100 (87.0)	
I don´t know	2 (0.9)	2 (2.0)	-

* Exact Fisher test

Chi^2^ Yates correction

**Table 4:** Practices related to COVID-19 in symptomatic and asymptomatic patients screened at the triage tent

Practices	Total n = 215 (100%)	Symptomatic n = 100 (46.5%)	Asymptomatic n = 115 (53.5%)	*p-value*
Face mask wear				
Which type of face mask do you wear?				0.151†
Fabric	134 (62.3)	69 (69.0)	6 (56.5)	
Surgical	75 (34.9)	29 (29.0)	46 (40.0)	
N95	6 (2.8)	2 (2.0)	4 (3.5)	
How often do you replace your face mask?				0.002†
Twice a day	8 (3.7)	2 (2.0)	6 (5.2)	
Daily	99 (46.0)	34 (34.0)	65 (56.5)	
Every two days	32 (14.9)	18 (18)	14 (12.2)	
Every 3 days or longer	76 (35.3)	46 (46.0)	30 (26.1)	
Hand washing				
How many times a day do you wash your hands with soap and water?				0.268†
1-2 times	14 (6.5)	9 (9.0)	5 (4.3)	
3-4 times	52 (24.2)	21 (21.0)	31 (27.0)	
5 times or more	148 (68.8)	69 (69.0)	79 (68.7)	
Never	1 (0.5)	1 (1.0)	-	
How many times a day do you clean your hands with alcohol gel?				0.065*
1-2 times	25 (11.6)	11 (11.0)	14 (12.2)	
3-4 times	30 (14.0)	9 (9.0)	21 (18.3)	
5 times or more	71 (33.0)	30 (30.0)	41 (35.7)	
Never	89 (41.4)	50 (50.0)	39 (33.9)	

* Chi^2^ Pearson

Exact Fisher test

As regards knowledge, we determined that disease transmission “by contact with droplets and secretions” (*p* = 0.021; adjusted Pearson: 0.307; Nagelkerke pseudo-R: 0.162) was related to the variable of educational level (*p* = 0.002) and found that patients with primary education were more likely to respond that COVID-19 is transmitted by droplets than those with other educational levels (B: 1.752; *p* = 0.004; OR: 5.765; 95% CI: 1.722-19.304). Likewise, regarding the origin of knowledge about COVID-19, the model (*p* < 0.001; adjusted Pearson: 0.644; Nagelkerke pseudo-R: 0.344) evidenced a relation (*p* = 0.038) between the occupation as lawyers, administrative workers, food sector workers, merchants, students, maintenance workers, and healthcare workers and obtaining the information from traditional sources while there was no relation between primary education level and obtaining information from traditional sources (table 5). Besides, we found a relationship between obtaining information from social networks (*p* < 0.001; adjusted Pearson: 0.106; Nagelkerke pseudo-R: 0.290) and age (*p* = 0.010). The younger the participant, the greater the probability that he/she obtained their knowledge from social networks while patients with only primary education (*p* < 0.001) were more likely to obtain their knowledge from social networks than those with other education levels. Regarding attitudes, the model (*p* = 0.006; adjusted Pearson: 0.996; Nagelkerke pseudo-R: 0.291) found a relationship between age and the belief that Venezuela is ready to control the virus (*p* = 0.037), with younger respondents being more likely to believe that this is possible (table 5).

**Table 5:** Socio-demographic characteristics and presence or absence of symptoms and their relationship with KAP

KAP	Beta	*p-value*	OR	95% confidence interval for OR	
Knowledge					
Transmission of the disease by contact with droplets and secretions					
from an infected person					
Educational level	1,752	0,004	5,765	1,722	
Source of information about COVID-19					19,304
Traditional media					
Occupation					
Lawyer	17,843	<0,001	56123332,1	7166647,9	
Administrative worker	17,536	<0,001	41277816,0	7708881,1	439512089,4
Food sector worker	18,380	<0,001	96014877,9	15354225,0	221025342,2
Merchant	17,752	<0,001	51231102,7	11274644,3	600411728,0
Student	18,850	<0,001	153622214,2	32676461,0	232790126,3
Maintenance	17,674	<0,001	47403428,4	7068080,3	722225846,1
Health care worker	18,669	<0,001	128179393,1	27088562,3	317920128,9
Primary schooling	-1,332	0,043	0,264	0,073	606527457,40,959
Social media networks					
Age	-0,034	0,035	0,967	0,937	0,998
Primary schooling	2,291	0,000	9,887	3,145	31,077
Attitudes					
Do you think Venezuela is prepared to control the virus (COVID-19)?	-0,033	0,012	0,967	0,942	0,993
Age					
Practices					
Frequent handwashing and primary schooling	2,444	0,048	11,57	11,02	129,37
Social distancing	2.036	<0,001	7,659	2,532	23,173
Primary schooling	1,194	0,019	3,3	1,217	8,959
Asymptomatic					
How frequently do you change your face mask?					
Occupation					
Healthcare worker (every 2 days)	-3,56	0,030	0,028	0,001	0,713
Merchant (every 2 days)	-2,97	0,033	0,051	0,003	0,781
Asymptomatic (daily)	1,96	0,001	7,1	2,3	21,9

Regarding practices, we found that frequent handwashing (*p* = 0.004; adjusted Pearson: 0.978; Nagelkerke pseudo-R: 0.539) was related to the educational level (*p* < 0.001), and in its turn, this was related to social distancing (*p* = 0.014; adjusted Pearson: 0.231; Nagelkerke pseudo-R: 0.237), as well as to the presence or absence of symptoms (*p* = 0.016) with asymptomatic patients being more likely to maintain social distance than symptomatic ones. The model was also valid in relating the frequency of changing the mask (*p* = 0.012; adjusted Pearson: 0.996; Nagelkerke pseudo-R: 0.409) with occupation (*p* = 0.049), as well as with the presence or absence of symptoms (*p* < 0.001). Patients working in the healthcare sector were less likely to change masks every two days, as well as merchants, while asymptomatic patients were more likely to change it daily (table 5).

## Discussion

We evaluated here the KAP towards COVID-19 in a sample of the Venezuelan population after the identification of the first case and before the beginning of the exponential increase of cases ^16^. Our results serve as a baseline report of the knowledge and practices of Venezuelan patients and suggest that patients who were screened in the HUC triage tent during the study period had adequate knowledge regarding COVID-19 symptoms, transmission, and preventive measures.

Unlike most KAP studies in other countries, which conducted online surveys through social networks ^8^^,^^14^^,^^17^^-^^21^, the data for this study was collected through face-to-face interviews with patients attending the HUC COVID-19 screening tent. This allowed us to collect a robust dataset about clinical symptoms and patients’ reasons for consultation. Interestingly, the majority of the surveyed patient population was asymptomatic and consulted to request a COVID-19 test (a rapid test) or a “no suspicion of COVID-19” report. This may be because Venezuela initiated quarantine protocols very quickly (four days after the first case) ^7^. National transit was rapidly restricted and international borders were temporarily closed, thus limiting local and national travel. Travelers and essential workers were required to report the results of screening tests to travel or return to work. However, this “immunological passport” could have given false security about not being infected because rapid tests have low sensitivity to detect infection in asymptomatic people or in the initial phase of the disease ^22^^,^^23^. This could have had consequences in patient behavior during the pandemic in the country.

Regarding the knowledge of COVID-19 symptoms, in other similar studies (14,18-20,24), most interviewees recognized fever, dry cough, and dyspnea as the main symptoms of a sick person. As in most countries, our results showed that the majority of interviewees considered using barrier methods (masks and gloves, handwashing, and social distancing) as the main preventive measures to avoid contagion (14,24). The participants obtained information on SARS-CoV-2 from several sources, television being the most prevalent (97.5%) followed by social networks (47.7%) such as Instagram® (50%), and instant messaging services such as WhatsApp® (47%). These results are consistent with other international studies ^21^. However, a preliminary systematic review of KAPs towards COVID-19 pandemic in America reviewing 13 articles from different areas of the American continent including eight from the United States, two from Brazil, one from Colombia, one from Paraguay, and one from Ecuador, found that people who used official government sites to get news and clarifications were associated with better knowledge about COVID-19 and were more likely to adhere to national health guidelines and show a positive attitude and correct behaviors. On the other hand, people who preferred information from social networks, besides having obvious knowledge gaps, were more prone to dangerous behaviors ^25^.

Concerning patients’ attitudes, the belief that the Venezuelan government could effectively control the virus (42.8%) was greater than in Perú (23.1%) ^11^; however, it was much less than in China (97.1%) and Tanzania (96%) ^14^^,^^19^, significantly inferior to other countries of the area, such as Colombia (51,3%), Paraguay (66%), and Ecuador (63%) ^26^^-^^28^, and similar to Brazil (41%) ^29^. Such results would suggest discontent and a high sense of risk among the surveyed population and be decisive for the development and application of public health measures to face the epidemic in the region. They would also explain why most surveyed patients agreed that quarantine protocols should continue in Venezuela.

Most participants practiced frequent handwashing (94.4%) and the use of face masks in public places (89.3%), even more frequently than reported in other countries such as Pakistan (88.1% and 85.8%, respectively), Malaysia (87.8% and 51.2%, respectively) ^8^^,^^24^, and Paraguay where only 74% reported using masks in public places ^27^. Our findings are similar to those reported in Colombia (94% and 89,3%, respectively) ^26^, probably due to the government’s campaign to broadcast information related to the disease and special measures like the mandatory use of face masks by citizens ^7^. However, both China and Ecuador report a broader use of face masks in public places (98% and 93%, respectively) ^14^^,^^28^. Most countries report individuals avoiding crowded places (96.4% in China, 93.7% in Australia, 90.4% in Iran, 90.3% in Nigeria, 89% in Ecuador, 97% in Colombia, 88% in Paraguay, among others) ^14^^,^^21^^,^^26^^-^^28^^,^^30^^,^^31^. While many of the surveyed participants frequently visited crowded places (56.3%), most of them had previously answered that they were respecting the quarantine (89.3%). This situation was also reported in Nigeria ^21^, where 27.5% of the population reported visits to crowded places during the period of the survey. This can be explained by the difficulty of local governments to implement strict prevention and control measures including the total closure of popular food markets, social distancing, and the mandatory use of face masks.

Additionally, most Venezuelan citizens rely on menial jobs for their daily survival, even during quarantine. The palliative measures provided by the government seemed to have been insufficient during the time of the survey. A similar situation was reported in Nairobi, Kenya, where most participants breached quarantine by returning to work due to dwindled income and food shortages ^18^. Finally, the economic crisis in Venezuela and the concomitant low salary make it more difficult for individuals to afford cleaning products or masks. This might explain the low frequency of alcohol-based gel solutions used for handwashing (41.4%) and the frequent use of face masks made from cloth (62.3%). Another factor that has to be taken under consideration is that although the population has good theoretical knowledge, this does not necessarily reflect in their behavior, as is the case of Brazil. A study in 4,436 individuals from different Brazilian regions showed that the population, in general, had satisfactory knowledge of COVID-19; however, the practice of social distancing in this country is still unsatisfactory, agglomeration cases are recurrent, and, although efficient, preventive measures do not show significant adherence by the population. Thus, there is a gap between theoretical knowledge and satisfactory practice ^29^.

Our multivariate analysis demonstrated that patients with primary education had more knowledge about SARS-CoV-2 transmission than other education levels. According to other studies on COVID-19, knowledge about the disease can increase among individuals from lower schooling levels when educational health programs are implemented ^14^, which could be related to our results. As in other studies ^(20, 21)^, knowledge in younger adults and patients with only primary education was probably acquired through social media, a popular new method reported worldwide. In contrast with results from another study ^32^, we found that among younger people positive attitudes associated with the belief that Venezuela is prepared for controlling the virus predominated, which could respond to the fact that young people are more optimistic about this matter. Regarding practices, the asymptomatic patients tended to be more inclined to wash their hands and avoid crowded places, with the exception of potential exposure at their workplaces, and handwashing with sanitizer was the most frequently used method, as was evidenced in other countries ^33^. In general, people with higher education levels tend to have better protection practices and asymptomatic patients tend to change their masks or disinfect them more frequently (at least every two days).

Although we provided key insight on patients’ KAPs about COVID-19 in Venezuela, our study has some limitations. First, the questionnaire was not previously validated for KAPs regarding COVID-19; however, the questions used were based on WHO recommendations and previous KAP studies validated in other countries ^13^^-^^15^, and second, we evaluated KAPs regarding COVID-19 in a single diagnostic center, so more studies are required to examine knowledge gaps and practices by patients treated at different satellite centers in the country. Notwithstanding these limitations, this is the first study evaluating KAPs regarding COVID-19 in Venezuela and our results can be used to promote healthy practices and enhance patient care through educational and preventive strategies at the national level. They could also be useful for public healthcare authorities from other countries in the American continent.

The study found adequate knowledge regarding the SARS-CoV-2 infection symptoms, transmission, and prevention among patients screened at the HUC triage tent, as well as high demand for the test by asymptomatic patients wanting to travel to other states or countries, or returning to work. The information was obtained during the early phase of the pandemic in Venezuela and could contribute to design COVID-19 prevention strategies, foster optimistic attitudes, and promote safe practices.
